# Supplementary material for: Smooth Interpolating Curves with Local Control and Monotone Alternating Curvature
Source: Comput Graph Forum. 2022 Oct 6;41(5):25–38. doi: 10.1111/cgf.14600 (PMC9827861; doi:10.1111/cgf.14600)
Supplement: Supplementary file 1 — Supplement Material [file CGF-41-25-s001.zip › Local-Smooth-Interpolating-MonoCurvature/extern/clothoids/docs/index.html]

G1 and G2 fitting with clothoids, spline of clothods, circle arc and biarc — Clothoids v2.0.9

### Navigation

- index
- toc
- next
- Clothoids »
- G1 and G2 fitting with clothoids, spline of clothods, circle arc and biarc

# G1 and G2 fitting with clothoids, spline of clothods, circle arc and biarc¶

**Github repository** https://github.com/ebertolazzi/Clothoids

**Matlab TOOLBOX** https://github.com/ebertolazzi/Clothoids/releases

## Installation¶

### Matlab¶

Download the toolbox and install as usual.
Run `CompileClothoidsLib` on Matlab console windows
to compile the `mex` of the library.

### C++¶

Download the library

```
git clone git@github.com:ebertolazzi/Clothoids.git —recurse-submodules
```

if you forget `—recurse-submodules` you must load the submdule
quarticRootsFlocke next. To compile the library you can use `make`

```
make
```

or `cmake`

```
mkdir build
cd build
cmake ..
make
```

of `rake`

```
rake build_osx   # on mac
rake build_linux # on linux
rake build_win   # on windows
```

using `make` you have the library and headers in the following tree

```
`-- lib
    |-- include
    |   |-- AABBtree.hh
    |   |-- Biarc.hh
    |   |-- BiarcList.hh
    |   |-- Circle.hh
    |   |-- Clothoid.hh
    |   |-- ClothoidAsyPlot.hh
    |   |-- ClothoidList.hh
    |   |-- Fresnel.hh
    |   |-- G2lib.hh
    |   |-- Line.hh
    |   |-- PolyLine.hh
    |   |-- PolynomialRoots-Utils.hh
    |   |-- PolynomialRoots.hh
    |   `-- Triangle2D.hh
    `-- lib
        |-- libClothoids_OSTYPE.dylib
        `-- libClothoids_OSTYPE_static.a
```

where OSTYPE can be `linux`, `osx`, `mingw_x64`, `win_x64`

## Contents¶

- Matlab Interface Manual
  - Installation mex/MATLAB interface
  - LineSegment
  - Constuctors
  - Methods
  - Evaluation Methods
  - CircleArc
  - Constuctors
  - Methods
  - Distance Methods
  - Evaluation Methods
  - Biarc
  - Constuctors
  - Methods
  - Distance Methods
  - Evaluation Methods
  - ClothoidCurve
  - Constuctors
  - Methods
  - Distance and intersection Methods
  - Evaluation Methods
  - ClothoidList
  - ClothoidSplineG2
  - Triangle2D
  - FresnelCS
- C++ API
  - Class Hierarchy
  - File Hierarchy
  - Full API
- MATLAB API
  - Class Hierarchy
  - File Hierarchy
  - Full API

### Authors¶

- Enrico Bertolazzi

  Dipartimento di Ingegneria Industriale

  Università degli Studi di Trento

  email:

  homepage: https://e.bertolazzi.dii.unitn.it
- Marco Frego

  Faculty of Science and Technology

  Free University of Bozen-Bolzano

  email:

### References¶

1. *E. Bertolazzi, M. Frego*, **G1 fitting with clothoids**,

   Mathematical Methods in the Applied Sciences,

   John Wiley & Sons, 2014, vol. 38, n.5, pp. 881-897,

   https://doi.org/10.1002/mma.3114
2. *E. Bertolazzi, M. Frego*, **On the G2 Hermite interpolation
   problem with clothoids**,

   Journal of Computational and Applied Mathematics,

   2018, vol. 15, n.341, pp. 99-116.

   https://doi.org/10.1016/j.cam.2018.03.029
3. *E. Bertolazzi, M. Frego*, **Interpolating clothoid splines with
   curvature continuity**,

   Mathematical Methods in the Applied Sciences,

   2018, vol. 41, n.4, pp. 1099-1476.

   https://doi.org/10.1002/mma.4700
4. *E. Bertolazzi, M. Frego* **Point-Clothoid Distance and Projection Computation**,

   SIAM Journal on Scientific Computing,

   2019, 41(5), A3326–A3353.

   https://doi.org/10.1137/18M1200439
5. *E. Bertolazzi, M. Frego* **A Note on Robust Biarc Computation**,

   Computer-Aided Design & Applications 16 (5), 822-835

   http://www.cad-journal.net/files/vol\_16/CAD\_16(5)\_2019\_822-835.pdf

### License¶

```
Copyright (c) 2020, Enrico Bertolazzi and Marco Frego
All rights reserved.

Redistribution and use in source and binary forms, with or without
modification, are permitted provided that the following conditions are
met:

    * Redistributions of source code must retain the above copyright
      notice, this list of conditions and the following disclaimer.
    * Redistributions in binary form must reproduce the above copyright
      notice, this list of conditions and the following disclaimer in
      the documentation and/or other materials provided with the distribution

THIS SOFTWARE IS PROVIDED BY THE COPYRIGHT HOLDERS AND CONTRIBUTORS "AS IS"
AND ANY EXPRESS OR IMPLIED WARRANTIES, INCLUDING, BUT NOT LIMITED TO, THE
IMPLIED WARRANTIES OF MERCHANTABILITY AND FITNESS FOR A PARTICULAR PURPOSE
ARE DISCLAIMED. IN NO EVENT SHALL THE COPYRIGHT OWNER OR CONTRIBUTORS BE
LIABLE FOR ANY DIRECT, INDIRECT, INCIDENTAL, SPECIAL, EXEMPLARY, OR
CONSEQUENTIAL DAMAGES (INCLUDING, BUT NOT LIMITED TO, PROCUREMENT OF
SUBSTITUTE GOODS OR SERVICES; LOSS OF USE, DATA, OR PROFITS; OR BUSINESS
INTERRUPTION) HOWEVER CAUSED AND ON ANY THEORY OF LIABILITY, WHETHER IN
CONTRACT, STRICT LIABILITY, OR TORT (INCLUDING NEGLIGENCE OR OTHERWISE)
ARISING IN ANY WAY OUT OF THE USE OF THIS SOFTWARE, EVEN IF ADVISED OF THE
POSSIBILITY OF SUCH DAMAGE.
```

### Quick search

### Table of Contents

- Matlab Interface Manual
- C++ API
- MATLAB API

«
hide menu

menu
sidebar
»

### Navigation

- index
- toc
- next
- Clothoids »
- G1 and G2 fitting with clothoids, spline of clothods, circle arc and biarc

© Copyright 2021, Enrico Bertolazzi and Marco Frego.
Created using Sphinx 4.2.0.
